# Hypoxia up-regulates SERPINB3 through HIF-2α in human liver cancer cells

**DOI:** 10.18632/oncotarget.2943

**Published:** 2014-12-10

**Authors:** Stefania Cannito, Cristian Turato, Claudia Paternostro, Alessandra Biasiolo, Sebastiano Colombatto, Irene Cambieri, Santina Quarta, Erica Novo, Elisabetta Morello, Gianmarco Villano, Silvano Fasolato, Tiziana Musso, Ezio David, Ignazia Tusa, Elisabetta Rovida, Riccardo Autelli, Antonina Smedile, Umberto Cillo, Patrizia Pontisso, Maurizio Parola

**Affiliations:** ^1^ Department of Clinical and Biological Sciences, Unit of Experimental Medicine and Interuniversity Center for Liver Pathophysiology, University of Torino, Italy; ^2^ Department of Medicine, University of Padova, Italy; ^3^ Department of Oncology, University of Torino, Italy; ^4^ Department of Plastic Surgery and Burn Unit Skin Bank, CTO Hospital, Torino, Italy; ^5^ Department of Public Health and Pediatric Sciences, University of Torino, Italy; ^6^ Pathology Unit, San Giovanni Battista Hospital, Torino, Italy; ^7^ Department of Biomedical, Experimental and Clinical Sciences “Mario Serio”, University of Firenze, Italy; ^8^ Gastroenterology and Hepatology Division, San Giovanni Battista Hospital, Torino, Italy; ^9^ Unit of Hepatobiliary Surgery and Liver Transplantation, University of Padova, Italy

**Keywords:** SERPINB3, hypoxia, hepatocellular carcinoma, HIF-2α, reactive oxygen species

## Abstract

SERPINB3 is a cysteine-proteases inhibitor up-regulated in a significant number of cirrhotic patients carrying hepatocellular carcinoma (HCC) and recently proposed as a prognostic marker for HCC early recurrence. SERPINB3 has been reported to stimulate proliferation, inhibit apoptosis and, similar to what reported for hypoxia, to trigger epithelial-to-mesenchymal transition (EMT) and increased invasiveness in liver cancer cells. This study has investigated whether SERPINB3 expression is regulated by hypoxia-related mechanisms in liver cancer cells.

Exposure of HepG2 and Huh7 cells to hypoxia up-regulated SERPINB3 transcription, protein synthesis and release in the extracellular medium. Hypoxia-dependent SERPINB3 up-regulation was selective (no change detected for SERPINB4) and operated through hypoxia inducible factor (HIF)-2α (not HIF-1α) binding to SERPINB3 promoter, as confirmed by chromatin immuno-precipitation assay and silencing experiments employing specific siRNAs. HIF-2α-mediated SERPINB3 up-regulation under hypoxic conditions required intracellular generation of ROS. Immuno-histochemistry (IHC) and transcript analysis, performed in human HCC specimens, revealed co-localization of the two proteins in liver cancer cells and the existence of a positive correlation between HIF-2α and SERPINB3 transcript levels, respectively.

Hypoxia, through HIF-2α-dependent and redox-sensitive mechanisms, up-regulates the transcription, synthesis and release of SERPINB3, a molecule with a high oncogenic potential.

## INTRODUCTION

Hypoxic areas, heterogeneously distributed within the neoplastic mass, represent a major and common feature of clinically relevant solid malignant tumors, including hepatocellular carcinoma (HCC) [1]. Hypoxia may develop as a consequence of poor/altered vascularization (perfusion-limited O_2_ delivery), deterioration of diffusion geometry (diffusion-limited O_2_ delivery) or to conditions of tumor- or therapy-associated anemia [1]. Although low oxygen tension may even contribute to kill some tumor cells, hypoxia is currently believed to provide a strong selective pressure able to regulate tumor growth and eventually favor proliferation and survival of the most aggressive malignant cells [1-6]. Along these lines, the detection of hypoxic areas within a neoplastic mass is considered as an independent prognostic indicator of poor outcome with a significant risk to develop metastasis that may escape therapy [1,3,6-8]. Indeed, neoplastic cells surviving hypoxia exhibit enhanced invasive propensity, suggesting that hypoxia may favor cancer progression [1-8]. Accordingly, hypoxia triggers epithelial-to-mesenchymal transition (EMT) in human cancer cells (including hepatic cancer cells) [9-11]. EMT is a differentiation and/or morphogenetic process involved in embryogenesis that has been proposed to contribute also to an increased invasiveness of cancer cells, cancer progression and metastasis [11-13].

Epidemiological analysis indicate that HCC, which develops usually in a cirrhotic background whatever the etiology (main risk factors being hepatitis B and C viral infections, alcohol abuse and metabolic syndrome), is at present one of the leading cause of cancer-related mortality worldwide. HCC has an extremely negative prognosis due to the fact that the majority of patients are diagnosed at an advanced stage, as well as to the lack of effective treatment, the high risk for tumor recurrence and the substantial lack of reliable markers predictive of recurrence [14,15]. In the last decade several studies have increased our knowledge on either the significance of molecular mechanisms of hepatic carcinogenesis, signal transduction pathways linked to HCC biology, then outlining potential therapeutic targets as well as molecular changes that may be useful as diagnostic or prognostic markers [16,17].

Along these lines, SERPINB3 a member of the ovalbumin-serine proteases inhibitor family (ov-serpins) [18], has been detected in several malignancies of epithelial origin, including HCC [19-23]. Concerning HCC, SERPINB3 and its isoform SERPINB4 (formerly known as squamous cell carcinoma antigen-1 or SCCA-1 and squamous cell carcinoma antigen-2 or SCCA-2, respectively), are undetectable in normal hepatocytes. However, their expression increases progressively in chronic liver diseases, in HCC cells and in cells of highly dysplastic nodules and hepatocytes of peri-tumoural cirrhotic tissue, suggesting that they may represent a relatively early event in hepatocarcinogenesis [21,24-26].

Indeed, although its precise *in vivo* role has not been identified yet, SERPINB3 has been reported *in vitro* to protect tumor cells from induction of apoptosis [27] and to induce epithelial-mesenchymal transition (EMT) and increased invasiveness as well as cell proliferation [28]. Moreover, SERPINB3/4 have been recently shown to be up-regulated by oncogenic Ras and to be able to promote NF-kB-related inflammatory cytokine production favoring tumor progression [23].

SERPINB3 has also been detected in the majority of hepatoblastomas, where the highest levels were found in tumors of more advanced stage [29]. Of interest, a very recent study performed in HCC specimens from surgically resected patients with adequate clinical follow-up revealed that high levels of SERPINB3 were detectable in 22% of HCC specimens and were found to be significantly associated with early tumor recurrence, then representing a subset of most aggressive HCCs [30].

However, we still ignore the nature of stimuli able to up-regulate SERPINB3 expression in chronic liver diseases and, in particular, in HCC. As mentioned earlier, in a previous study we reported that SERPINB3 was able to trigger EMT and increased invasiveness in HepG2 cells and human hepatocytes, possibly acting as a paracrine/autocrine mediator [28]. EMT induction triggered by SERPINB3, in particular, closely resembled the scenario observed by us [10] and others [11,31] in cancer cells of different origin exposed to hypoxic conditions, with hypoxia-induced EMT found to involve hypoxia-inducible factors (HIFs), a family of heterodimeric transcription factors acting as master regulators of homeostatic responses to low oxygen tension [5-8]. Hypoxic areas are commonly detected in HCC specimens and a preliminary gene data analysis revealed that the consensus core HRE (hypoxia-responsive element) RCGTG sequences are present at the SERPINB3 promoter (Supplemental Figure 1) [32].

In the present study, performed on human HCC cell lines and HCC specimens, we report for the first time that SERPINB3 is up-regulated by hypoxic conditions through a selective HIF-2α-dependent mechanism in liver cancer cells and then released in a paracrine way. In keeping with these findings, a positive correlation between HIF-2α and SERPINB3 was detected at transcript and protein level in HCC specimens. In particular, the sub-population of patients with higher levels of transcripts for both molecules carried the most aggressive form of HCC, with early tumor recurrence.

## RESULTS

### SERPINB3 expression in liver cancer cells is up-regulated by hypoxia

**Figure 1:**
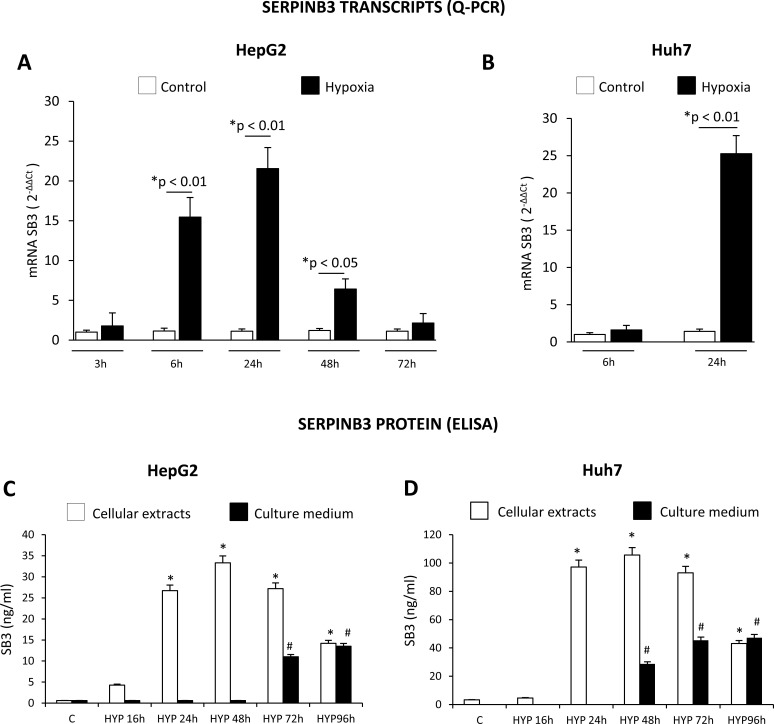
Hypoxic conditions up-regulate SERPINB3 (SB3) expression Panels A,B. Time-dependent analysis of SERPINB3 transcripts by quantitative real-time PCR (Q-PCR) in HepG2 (panel A) or Huh7 (panel B) cells not exposed (control, C) or exposed to hypoxic conditions (HYP) for the indicated times (*p< 0.05 vs control values of SB3; **p< 0.01 vs control values of SB3). Panels C,D. Time-dependent analysis of SERPINB3 protein levels (ng/ml) by ELISA in cellular extracts (white columns) or culture medium (black columns) of HepG2 (panel C) or Huh7 (panel D) cells not exposed (control, C) or exposed to hypoxia (HYP) for the indicated times. Data are expressed as means ± SEM of three independent experiments (*p< 0.01 vs control values of SB3 in cellular extracts; ^#^p< 0.01 vs control values of SB3 release in culture medium).

In order to identify a possible link between hypoxia and SERPINB3 expression we performed immuno-histochemistry analyses on serial sections obtained from a series of human HCC specimens (n=18) developed in cirrhotic livers (HCV etiology, G1 and G2 grading) and positive for SERPINB3. This preliminary analysis, showed that SERPINB3 (SB3) immuno-positivity was detectable in the same cells and areas positive for either HIF-1α and VEGF, supporting the hypothesis of a correlation between hypoxia and SERPINB3 expression (Supplemental Figure 2A,B). We therefore started to investigate the link between hypoxia and SERPINB3 expression by performing a first series of experiments in which two human liver cancer cell lines (HepG2 and Huh7 cells) were initially exposed to moderate hypoxia (3% O_2_) for up to 96 hrs. Under these experimental conditions HepG2 and Huh7 cells showed no significant signs of cell death, either necrotic or apoptotic, as already previously reported [10]. Exposure to hypoxia (3% O_2_) resulted in a significant up-regulation of SERPINB3 mRNA transcription, as quantified by Q-PCR (Figure 1A,B), that was apparently earlier in HepG2 cells (6 hr) than in Huh7. In HepG2, hypoxia-induced SERPINB3 transcription was maximal at 24 hrs and declined at later time points (Figure 1A). In preliminary experiments exposure of HepG2 to more severe conditions of hypoxia (0.1% O_2_) still resulted in early up-regulation of SERPINB3 transcripts (1 and 3 hrs, Supplemental Figure 3) but also in subsequent induction of significant cell death (data not shown). Whether the synthesis of the protein is concerned, as determined by ELISA, exposure of both HepG2 and Huh7 cells to hypoxia (3% O2) similarly induced (Figure 1C,D): a) a marked increase in intracellular SERPINB3 protein levels, that became detectable after 24 hrs of incubation, lasted until 72 hrs to decline at 96 hrs time point; b) a marked and delayed release of SERPINB3 in the extracellular medium that became detectable after 72 hrs (HepG2 cells) or 48 hrs (Huh7 cells). Up-regulation of SERPINB3 mRNA levels and protein synthesis as well as the delayed release of SERPINB3 protein in the extracellular medium was also detected in HT-29 human colon cancer cells exposed to identical conditions of hypoxia (Supplemental Figure 4A,B), suggesting that hypoxia-dependent up-regulation of SERPINB3 may be not limited to hepatic cancer cells. On the other hand, further experiments showed that hypoxia-induced up-regulation of SERPINB3 is a selective response since no significant change in transcript levels for SERPINB4 (i.e., a serine-protease inhibitor known to share a high degree of homology with SERPINB3 [21,23-26]) was detected in either HepG2 or HuH7 cells exposed to hypoxic conditions (Suppl. Figure 5A,B).

### Hypoxia-induced SERPINB3 up-regulation is dependent on HIF-2α

**Figure 2:**
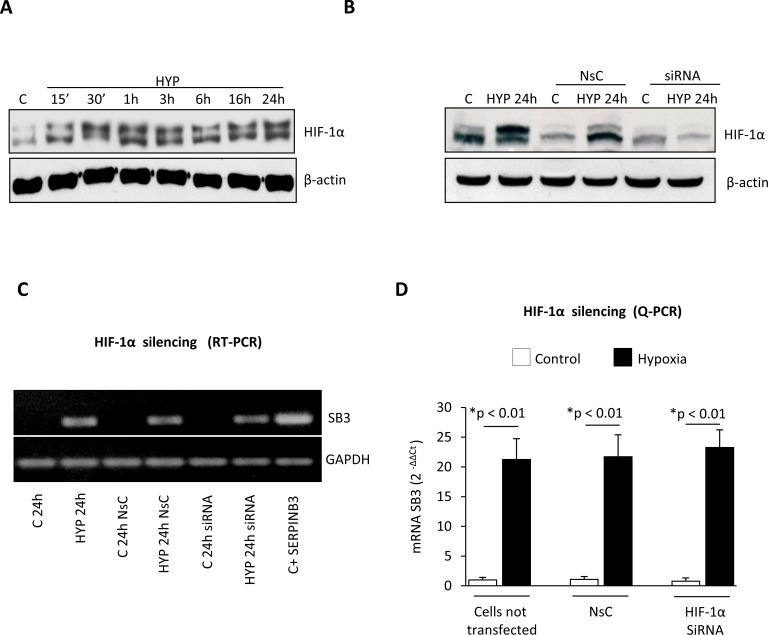
Hypoxia-dependent SERPINB3 (SB3) up-regulation is unrelated to HIF-1α Panel A. Western blot (WB) analysis of HIF-1α performed on total extracts of HepG2 cells (equal loading monitored by re-blotting membranes for β-actin) that were maintained in normoxic conditions (control, C) or exposed to hypoxia (HYP) for the indicated times. Panel B. WB analysis of HIF-1α expression in HepG2 cells not transfected, transfected with non-silencing control (NsC) or transfected with HIF-1α siRNA that were maintained in normoxic conditions (control, C) or exposed to hypoxia for 24 hrs. Panel C, D. Analysis of SERPINB3 transcripts by RT-PCR (panel C) or Q-PCR (panel D) in HepG2 cells treated as in (B) or as indicated.

**Figure 3:**
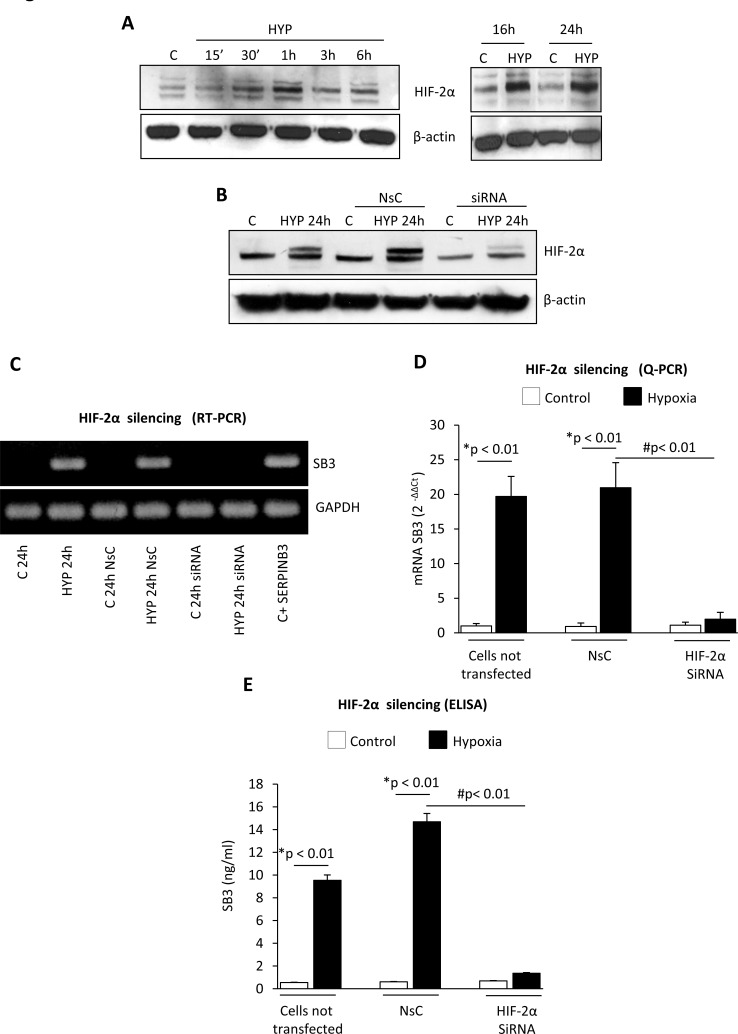
Hypoxia-dependent SERPINB3 (SB3) up-regulation involves HIF-2α Panel A. WB analysis of HIF-2α protein levels performed on total extracts of HepG2 cells (equal loading monitored by re-blotting membranes for β-actin) that were maintained in normoxic conditions (control, C) or exposed to hypoxia (HYP) for the indicated times. Panel B. WB analysis of HIF-2α expression in HepG2 cells not transfected, transfected with non-silencing control (NsC) or transfected with HIF-2α siRNA that were maintained in normoxic conditions (control, C) or exposed to hypoxia for 24 hrs. Panels C, D. Analysis of SERPINB3 transcripts by RT-PCR (panel C) or Q-PCR (panel D) in HepG2 cells treated as in (B) or as indicated. Panel E. ELISA analysis of intracellular SERPINB3 protein levels (ng/ml) in HepG2 cells treated as mentioned in (B-D). Data are expressed as means ± SEM of three independent experiments (* p<0.01 vs control values; ^#^ p<0.01 vs related treatment condition).

HIF-1α protein, a master regulator of hypoxia-induced responses, is increased in HepG2 cells exposed to hypoxia (Figure 2A) and consensus core HRE (hypoxia-responsive element) RCGTG sequences are present at the SERPINB3 promoter (Supplemental Figure 1) [32]. However, effective silencing of HIF-1α (evaluated as protein levels, Figure 2B) in HepG2 cells exposed to hypoxia by using a specific siRNA [10] did not prevent hypoxia-induced increase of SERPINB3 mRNA levels after 24 hrs of hypoxia (i.e., the time point with SERPINB3 peak transcription), as verified by RT-PCR (Figure 2C) and quantified by Q-PCR (Figure 2D). Since SERPINB3 promoter has, close to HRE sequences, one CGGA and four GGAC sequences (Supplemental Figure 1), which are ETS sites proposed to increase HIF-2α (not HIF-1α) binding affinity, [33,34] we then analyzed the possible involvement of HIF-2α. A time course analysis showed that hypoxia induced an early (30-60 min) and sustained (up to 24 hrs) increase of HIF-2α in HepG2 cells (Figure 3A). Moreover, following efficient silencing of HIF-2α with siRNA (Figure 3B), SERPINB3 transcripts, as verified by RT-PCR (Figure 3C) and quantified by Q-PCR (Figure 3D), as well as intracellular SERPINB3 protein levels (Figure 3E) were almost completely abolished in HepG2 cells exposed to hypoxia (24 hrs) as compared with related controls.

**Figure 4:**
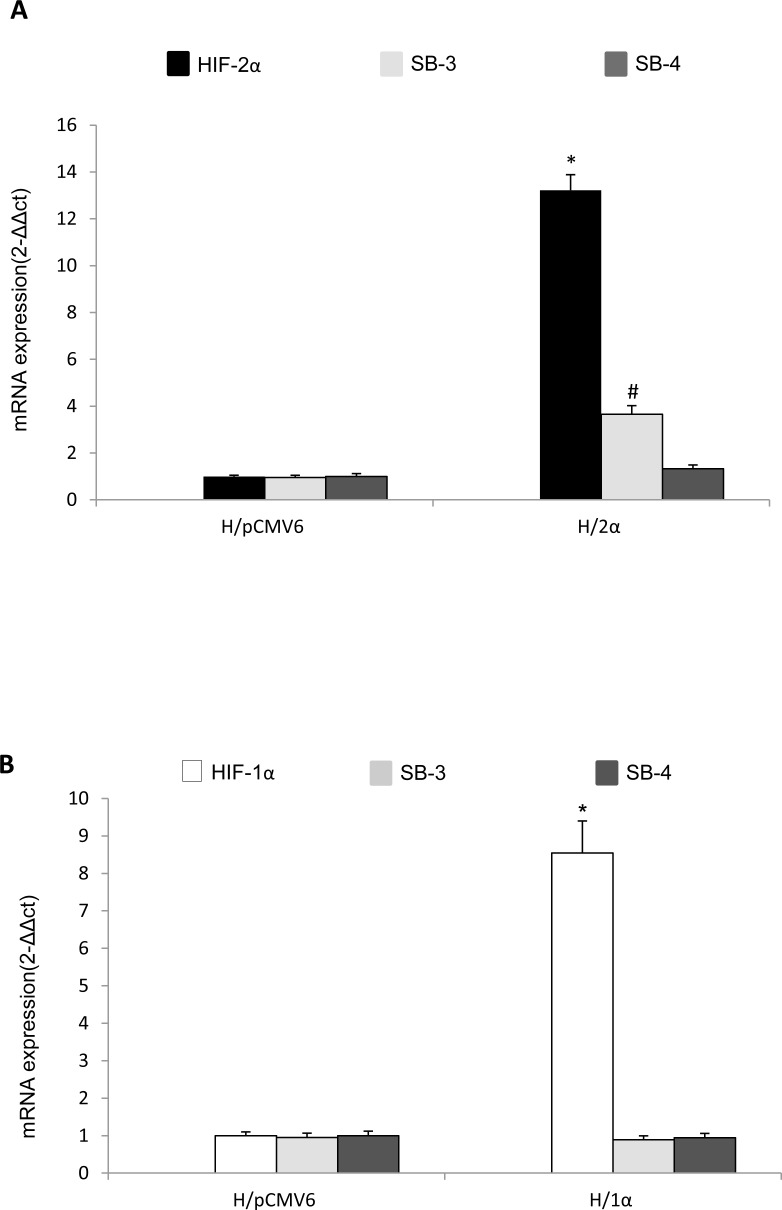
Selective relationships between HIF-2α and SERPINB3 up-regulation A,B. Analysis of SERPINB3, SERPINB4 and HIF-1α or HIF-2α transcripts by quantitative real-time PCR (Q-PCR) in HepG2 normoxic cells stably transfected in order to over-express either HIF-2α (H/2α, panel A) or HIF-1α (H/1α, panel B) (*p< 0.01 vs control values of transcripts in HepG2 cells transfected with empty vector, H/pCMV6).

In order to confirm the selective cause-effect relationships between HIF-2α and SERPINB3 up-regulation we performed further experiments in HepG2 cells stably transfected in order to over-express either HIF-1α (H/1α) or HIF-2α (H/2α) (Figure 4A,B). As evaluated by Q-PCR only HepG2 cells genetically manipulated to over-express HIF-2α also showed up-regulation of SERPINB3 mRNA levels, but not of SERPINB4, confirming the selectivity of the relationships.

### HIF-2α binds to SERPINB3 promoter

**Figure 5:**
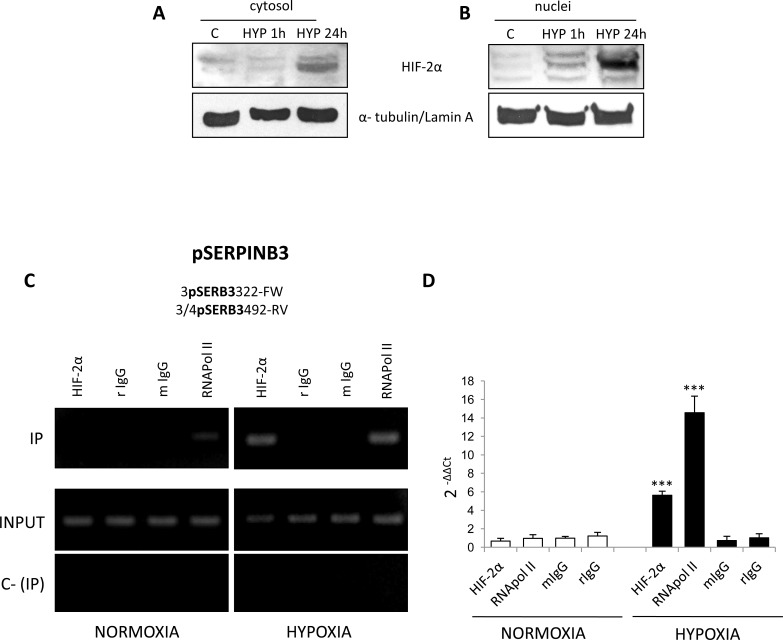
Hypoxia induces HIF-2α nuclear translocation and its binding to SERPINB3 promoter Panels A,B. WB analysis of HIF-2α in cytosolic (panel A) or nuclear (panel B) fractions obtained from HepG2 cells maintained in normoxic conditions (control, C) or exposed for 1 hr or 24 hrs to hypoxia (HYP); equal loading evaluated by re-blotting membranes for α-tubulin or lamin A for cytosol and nuclear fractions, respectively. Panels C,D. HepG2 cells were incubated for 3 hrs in normoxia or hypoxia and lysed. Lysates were subjected to ChIP using antibodies against HIF-2α, RNApol II or control IgG (rabbit, r; mouse, m). Panel C. RT-PCR for SB3 promoter (pSERPINB3) was performed with IPed DNA after IP or using input DNA (INPUT). C- (IP) was a negative control in the absence of antibody. Images obtained in one experiment out of three which provided similar results. Panel D. Q-PCR for SB3 promoter. Histograms represent the relative quantification of DNA recovered from IP with the indicated antibodies. Values were intra-experimentally normalized for input DNA and control IgG. Data represent the average ± SD of one experiment out of two performed in triplicate. The statistical significance of differences was determined by the Student's t-test (*** p < 0.001).

Relationships between hypoxia, HIF-2α and SERPINB3, were further investigated on cytosolic and nuclear fractions obtained from HepG2 exposed to hypoxia (Figure 5A,B). A significant increase in nuclear HIF-2α protein levels was already detectable after 1 hr and then strongly enhanced after 24 hrs. By performing a ChIP assay in HepG2 cells we found that, as verified by RT-PCR (Figure 5C) and quantified by Q-PCR (Figure 5D) in normoxic HepG2 cells HIF-2α was not bound to SERPINB3 promoter. In keeping with the absence of expression of SERPINB3 in control cells and low recruitment of RNA polymerase type II (RNApol II) at SERPINB3 promoter in normoxia, this indicates that transcription at this site is inactive under normal levels of oxygen. Following 3 hrs of exposure to hypoxia, HIF-2α and RNApol II bound to SERPINB3 promoter in keeping with the expression of SERPINB3 mRNA. Additional ChIP assay performed in the same experimental conditions excluded the binding of HIF-1α to SERPINB3 promoter (Supplemental Figure 6), confirming the possible involvement of HIF-2α in hypoxia–induced SERPINB3 expression.

### Hypoxia-induced SERPINB3 up-regulation involves intracellular generation of ROS

**Figure 6:**
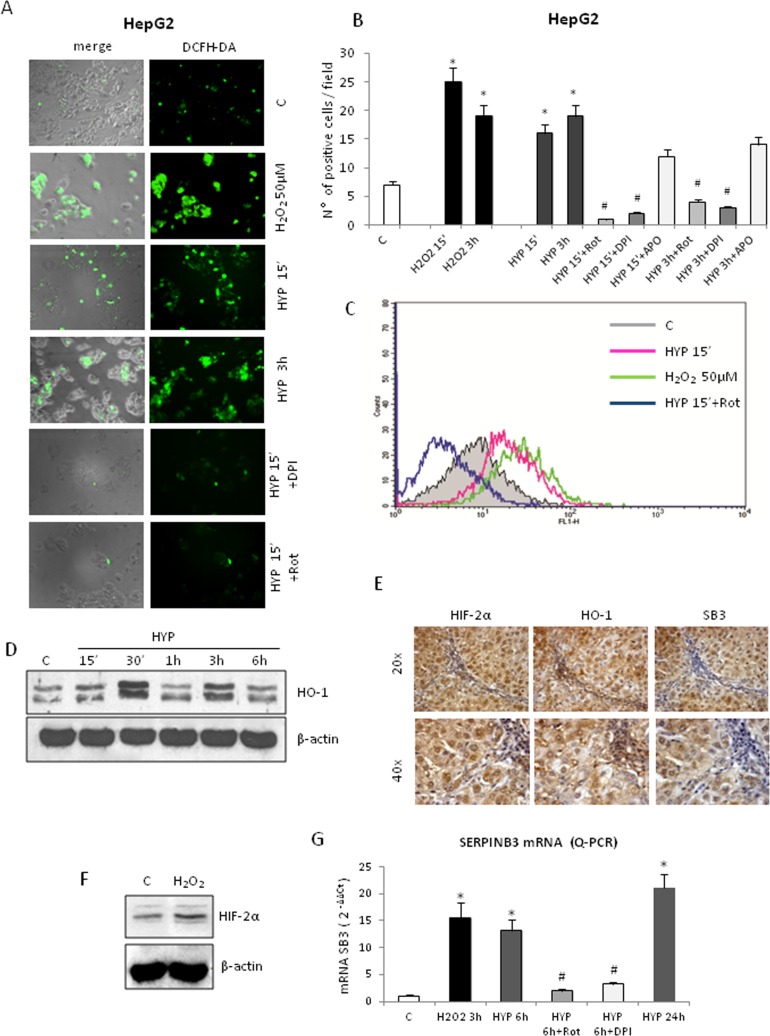
Hypoxia-dependent SERPINB3 (SB3) up-regulation involves intracellular ROS generation Panels A,B,C. Detection of intracellular ROS (DCFH-DA probe) in HepG2 cells not exposed (control, C), exposed to H_2_O_2_ 50μM or exposed to hypoxia (HYP) for the indicated times detected using fluorescence microscope (panel A, DCFH-DA fluorescence; panel B, number of positive cells for field, means ± SEM of three different experiments; * and # p < 0,01 vs control values or related treatment condition, respectively) or by employing flow cytometric analysis (panel C). In some experiments HepG2 cells were pre-treated with Rotenone (Rot), DPI or Apocynin (APO). (Panel D). WB analysis of HO-1 protein levels on total extracts obtained from HepG2 cells maintained in normoxic conditions (control, C) or exposed to hypoxia up to 6 hrs (HYP) (equal loading monitored by reblotting membranes for β-actin). Panel E. IHC analysis on serial sections obtained from human SERPINB3 positive HCC (grading G2) developed in a cirrhotic patient, showing that expression of SB3 and HIF2-α is detectable in the same areas also positive for HO-1. Panel F. WB analysis of HIF-2α recruitment/stabilization in HepG2 cells treated with H_2_O_2_ 50μM. Panel G. Q-PCR analysis of SB3 transcripts in HepG2 cells not exposed (control, C), or exposed to H_2_O_2_ or hypoxia (HYP) up to 24hrs. In some conditions cells were pre-treated with Rot or DPI.

We previously showed that ROS of mitochondrial origin are produced in a number of cell lines (including HepG2 cells) following exposure to hypoxia and that these ROS can affect HIF1-α recruitment/stabilization, [10] in line with established literature evidence [35]. Exposure of HepG2 cells to hypoxia resulted in an early (15 minutes, Figure 6A-C) and transient (sustained up to 3 hrs, declining afterward) increase in intracellular ROS (DCFH-DA technique, hydrogen peroxide or H_2_O_2_ used as positive control). This effect was observed also in Huh7 cells (Supplemental Figure 7) and was almost completely abolished by pre-treating HepG2 cells with either the mitochondrial electron chain inhibitor rotenone or the more general inhibitor of flavin-dependent enzymes DPI (Figure 6A,B). Rotenone is known to block hypoxia-related mitochondrial release of ROS whereas DPI can also block NADPH-oxidase dependent generation of ROS, which has also been suggested to play a role in hypoxic conditions. However, the use of the more specific inhibitor of NADPH-oxidase activity apocynin (Figure 6B) was unable to significantly prevent hypoxia-dependent ROS generation in our experimental conditions, suggesting that intracellular ROS generated under our conditions of hypoxia should be mainly of mitochondrial origin. Identical results (i.e., early ROS increase following hypoxia, abolished by short pre-treatment with rotenone) were obtained in experiments in which intracellular ROS generation was measured by DCFH-DA technique combined to cytofluorimetric analysis (Figure 6C). Intracellular ROS generation was also confirmed by the rapid increase of heme oxygenase-1 (HO-1) protein levels, a well established early “sensor” of intracellular redox changes [36], as shown *in vitro* in HepG2 cells (Figure 6D). Accordingly, immuno-histochemistry analysis performed in serial sections from human HCC tissue positive for SERPINB3 (Figure 6E), showed positive staining in the same areas/cells also for HO-1 and HIF-2α. Moreover, we found that exposure of HepG2 cells to H_2_O_2_ resulted in an early (1 hour) recruitment/stabilization of HIF-2α (Figure 6F), as already described for HIF-1α [35]. Accordingly, pre-treatment with either rotenone or DPI prevented hypoxia-induced SERPINB3 transcription at 6 hrs, as quantified by Q-PCR (Figure 6G), indicating the involvement of ROS in this phenomenon. This finding is fully in agreement with previously published data suggesting that hypoxia- and mitochondria-derived ROS act likely by affecting prolyl-hydroxylases [35]. Indeed, SERPINB3 mRNA was significantly and rapidly up-regulated in HepG2 normoxic cells following exposure to H_2_O_2_, suggesting that hypoxia-related transient increase in intracellular ROS represents a critical early event mediating SERPINB3 up-regulation (Figure 6G).

### SERPINB3 and HIF-2α expression in human HCC specimens

**Figure 7:**
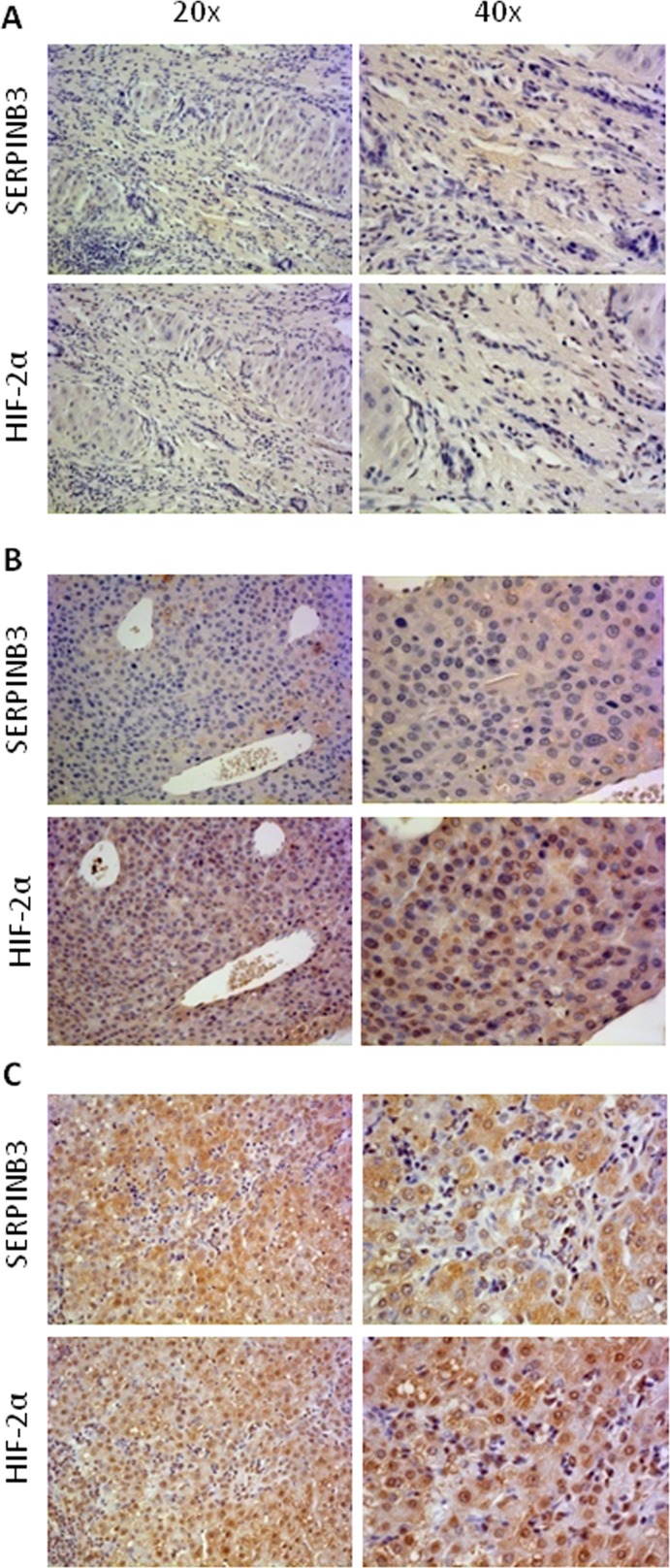

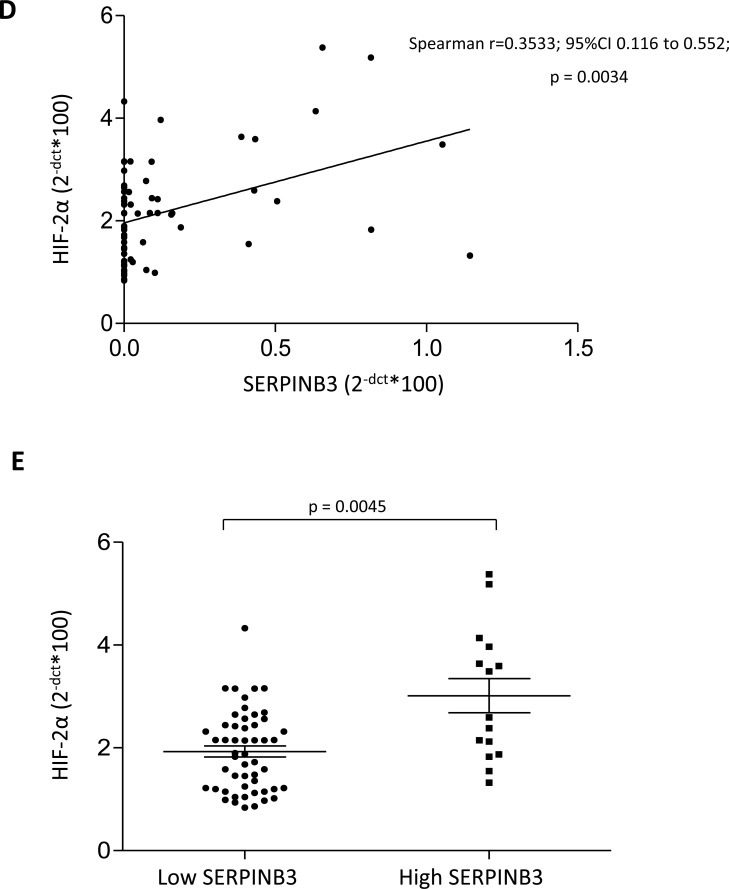
“*In vivo*” evidence for HIF-2α and SERPINB3 (SB3) correlation in human HCC Panels A-C. IHC analysis on serial sections of human HCC from cirrhotic patients carrying HCC showing different patterns of expression of SERPINB3 and HIF-2α: pattern 1, negative for either SERPINB3 or HIF-2α (panel A); pattern 2, weakly positive for SERPINB3 and positive, mainly cytoplasmic, staining for HIF-2α (panel B); pattern 3, intense positive stain for both SERPINB3 and HIF-2α, the latter showing mainly nuclear staining (panel C). Original magnification as indicated. Panel D. SERPINB3 and HIF-2α mRNA were analysed by Q-PCR in HCC specimens obtained from a cohort of 67 patients. Statistical analysis was performed using Spearman correlation. Panel E. SERPINB3 and HIF-2α mRNA were analysed by Q-PCR in relation to low expression (SERPINB3 low; N=52) or high expression (SERPINB3 high; N=15) of SERPINB3 mRNA. The y axis represents the relative mRNA amounts of the normalised gene. Central bars represent mean and external bars represent s.e.m. The analysis was performed with Mann-Whitney test.

In a first series of experiments, designed to investigate a possible relationship between HIF-2α and SERPINB3 expression directly on HCC tissue, we performed immuno-histochemistry (IHC) on paraffin-included human liver specimens (n=18). These analysis offered an overall morphological scenario in which human samples were characterized by either i) negativity for SERPINB3 and negativity or low/moderately positivity for HIF-2α (group 1: 9 patients out of 18, Figure 7A), ii) low/moderate SERPINB3 positivity associated with more intense staining for HIF-2α (group 2: 5 patients out of 18, Figure 7B), iii) intense positive staining for both SERPINB3 and for nuclear HIF-2α (group 3: 4 out of 18 patients, Figure 7C). Along these lines, in a recent human study [30] some of us reported that high levels of SERPINB3 transcripts were detectable in 15 out of 67 human HCC specimens (approx. 22%) and that high expression levels of SERPINB3 were significantly associated with early tumor recurrence. In order to further verify the significance of the correlation between HIF-2α and SERPINB3 we performed Q-PCR analysis of both HIF-2α and SERPINB3 transcripts in human HCC specimens obtained from the same cohort of 67 well characterized patients recently described [30]. Statistical analysis extended to all available specimens revealed the existence of a positive correlation (Spearman r coefficient, p < 0.01) between HIF-2α and SERPINB3 transcript levels (Figure 7D), supporting the notion that also *in vivo* HIF-2α may play a relevant role in increasing SERPINB3 expression. In agreement with these findings, the highest levels of HIF-2α transcription were found in the sub-class of patients with high (> median value) SERPINB3 expression (Figure 7E) and previously described to carry the higher rate of early tumor recurrence [30].

## DISCUSSION

In a previous study we reported that SERPINB3 was able to induce EMT-related changes and increased invasiveness in HepG2 and MDCK cells [28]. Moreover, high levels of SERPINB3 expression were detected in the sub-class of HCCs with poor prognosis [30]. Here we provide evidence for the first time indicating that SERPINB3 expression is significantly and mechanistically up-regulated by hypoxia through HIF-2α-dependent mechanisms in human liver cancer cells. Of relevance, in human HCC specimens the expression of SERPINB3 and HIF-2α transcripts was significantly correlated, with high levels of both transcripts being found in HCC specimens from patients that experienced early HCC recurrence.

As previously established for HIF-1α, [35,37,38] our data indicate that HIF-2α-dependent up-regulation of SERPINB3 requires generation of ROS (apparently of prevalent mitochondrial origin) and redox-mediated signaling. This is based on the following evidence: a) the rise in intracellular ROS is a very early (i.e., within 15 minutes) and transient event (declining after 3 hrs) that precedes and parallels HIF-2α recruitment/stabilization, its binding to SERPINB3 promoter and subsequent SERPINB3 mRNA transcription; b) the early rise of both ROS and SERPINB3 mRNA levels was abolished by pre-treating cells with rotenone, a pharmacological inhibitor of mitochondrial electron transport chain known to block hypoxia-induced mitochondrial release of ROS and HIF-1α and HIF-2α recruitment/stabilization; c) treatment of normoxic cells with H_2_O_2_, which is known to induce recruitment/stabilization of HIF-1α, can also induce recruitment/stabilization of HIF-2α and the rise of SERPINB3 mRNA. Moreover, images from human HCC specimens indicate that positive stain for HO-1, a protein which is known to be expressed in a redox-sensitive way in tissues undergoing generation of ROS [36,39], was detected in the same tumor areas positive for HIF-2α and SERPINB3. Results obtained in our laboratory demonstrate that hypoxia-dependent and ROS-related up-regulation of SERPINB3 involves activation and phosphorylation of Ras/ERK signaling cascade, since the pre-treatment of hypoxic HepG2 cells with pharmacological inhibitor of MEK (PD 98059) completely abolished SERPINB3 expression (Supplemental Figure 8). These data are consistent with the results reported by Catanzaro et al. which mechanistically demonstrated that Ras-induced up-regulation of SERPINB3/4 is dependent on MAPK pathway and ERK phosphorylation [23].

ROS likely act by inhibiting prolyl hydroxylases, [35,37,38] thus preventing HIF-α subunits hydroxylation, binding to VHL, ubiquitylation and proteasome degradation.

Selective involvement of HIF-2α recruitment/stabilization in hypoxia-dependent SERPINB3 up-regulation was unequivocally established by mechanistic experiments indicating that both increased transcription of SERPINB3 mRNA and SERPINB3 protein release in the extracellular medium were abolished following silencing of HIF-2α but not of HIF-1α. Moreover, only HepG2 genetically manipulated in order to over-express HIF-2α showed increased transcription of SERPINB3 mRNA levels. Data from ChIP assays revealed that HIF-2α (not HIF-1α) binds to SERPINB3 promoter very early (3 hrs) after exposure to hypoxia and that this is paralleled by the binding of RNApol II, indicating that transcription of SERPINB3 gene (silent under normoxic condition) is active following exposure to hypoxia. HIF-2α binding to SERPINB3 promoter occurs (on the basis of primers designed to perform ChIP assay) at the level of a region containing two CGTG HRE consensus sequences as well as the GGAC ETS site that are known to increase HIF-2α binding activity [33,34]. This fact may well explain why HIF-1α does not bind to SERPINB3 promoter following exposure to hypoxia. In addition, it should be noted that SERPINB3 up-regulation under conditions of hypoxia is a selective event since in the same experimental conditions no change was detected in the expression of SERPINB4, a serine-protease inhibitor sharing a high degree of homology with SERPINB3 [21,23-26].

This study unequivocally links SERPINB3 up-regulation to hypoxia through the action of HIF-2α, not HIF-1α. This concept is further supported by either immuno-histochemistry analysis performed in a series of HCC specimens and by the analysis of SERPINB3 and HIF-2α transcript levels in HCC specimens obtained from a well characterized cohort of 67 HCC patients [30]. Indeed, statistical analysis performed on the latter cohort revealed the existence of a positive linear correlation between SERPINB3 and HIF-2α transcripts in HCC specimens from such patients, and the highest levels of HIF-2α were found in the subgroup of patients with high SERPINB3, previously characterized for early tumor recurrence and poor prognosis. These findings suggest that HIF-2α expression is likely to play a role in such a negative clinical outcome.

Along these lines, literature indicates that HIFs, detected in most solid tumors, can be considered as negative prognostic factors predictive for a worst outcome, irrespectively of therapeutic treatment [1-8]. HIF-activated pathways and target genes, including VEGF-A and carbonic anhydrase IX, are believed to significantly contribute to the survival and progression of cancer cells, with therapeutic targeting of HIFs emerging as a rational approach to treat solid tumors [3,5,6]. Although HIF-1α and HIF-2α share a high degree of sequence identity, the ability to hetero-dimerize with ARNT and to bind to HRE sequences in order to activate transcription of common genes (including VEGF-A, GLUT1 and ADM-1), relevant differences have emerged for these two transcription factors [6,8,40,41]. HIF-1α is expressed ubiquitously whereas HIF-2α expression is restricted to vascular endothelial cells, neural crest-derived cells, type II pneumocytes, hepatocytes, cardiomyocytes and interstitial kidney cells [8,42,43]. Moreover, HIF-2α (not HIF-1α) up-regulates transforming growth factor-α and cyclin D1 genes as well as c-Myc activity and its overexpression in human tumors has been described to correlate with a faster rate of neoplastic cell growth [8,44,45]. HIF-2α, at least in some cancers, has a greater oncogenic capacity than HIF-1α and its overexpression (and that of HIF-1α) correlates with poor patient outcome in colorectal carcinoma, melanoma, ovarian cancer and hepatocellular carcinoma, [8,30,44-46] possibly by promoting Myc activity, and radio- and chemo-resistance through indirect suppression of p53 activity [8,44-46]. In this connection, HIF-2α may act through up-regulation of KLF4, Sox2 and Octamer-4, to favor de-differentiation and confirmation of stem-cell properties of putative cancer stem cells [8,11,31].

Finally, the emerging relationships among hypoxia, HIF-2α and SERPINB3 become particularly intriguing on the basis of the proposed hypothesis of a switch from HIF-1 to HIF-2 (and then from HIF-1α to HIF-2α) - dependent transcription during chronic hypoxia in solid tumors, favoring involvement of cancer stem cells and promoting inhibition of apoptosis, induction of cell proliferation and invasiveness/metastasis [47,48]. Indeed, although a single report has proposed a possible tumor-suppressor role for HIF-2α in HCC cells, [49] hypoxia and HIFs (HIF-1α and HIF-2α) are currently seen as significant determinants of the progression of chronic liver diseases and HCC development [49]. Remarkably, SERPINB3, which has been identified in the present study as a HIF-2α specific target, has been reported to be able to inhibit apoptosis and to stimulate proliferation (through c-Myc activation) as well as EMT and increased invasiveness in cancer cells [27-29], features that fit well with the recently proposed prognostic relevance of SERPINB3 whose expression has been recently reported to be significantly associated with early tumour recurrence and worse clinical outcome [30]. Along these lines, preliminary experiments (Supplemental Figure 9), performed in normoxic cells exposed to hypoxia-conditioned medium (CM-HYP) collected at 96 hrs from HepG2 exposed to hypoxia (i.e., a medium containing high levels of hypoxia-induced SERPINB3 protein), indicated that the use of a specific SERPINB3 neutralizing antibody resulted in a significant inhibition of CM-HYP-induced proliferation (evaluated as PCNA expression, Supplemental Figure 9A) as well as to a less extent, CM-HYP-induced invasiveness (Matrigel invasion assay, 9B). These preliminary “*in vitro*” data may suggest that SERPINB3 up-regulated by hypoxia may have a role in sustaining proliferation and invasiveness of hepatic cancer cells, both effects already reported in previous studies performed using recombinant human SERPINB3 or cells overexpressing SERPINB3 [28]. Further studies properly designed are necessary to fully confirm this hypothesis.

## MATERIALS AND METHOD

### Materials

Enhanced chemiluminescence (ECL) reagents, nitrocellulose membranes (Hybond-C extra), and secondary Cy3-conjugated antibodies were from Amersham Pharmacia Biotech Inc. (Piscataway, NJ, USA). Monoclonal antibody against SERPINB3 (sc-21767), LaminA (sc-20680) and PCNA (sc-25280) or polyclonal antibody for HIF-1α (sc-53546) were from Santa Cruz Biotechnology (Santa Cruz, CA, USA). Polyclonal antibody for HIF-2α and HO-1 were from Novus Biologicals (Cambridge, UK) and Stressgen (Victoria, BC, Canada), respectively. Monoclonal antibodies for α-tubulin and β-actin and all other reagents of analytical grade were from Sigma Chemical Co (Sigma Aldrich Spa, Milan, Italy), Lipofectamine 2000 (Invitrogen-Life Technologies), Plasmid DNA purification NucleoBond XtraMIDI (Macherey-Nagel, Germany), pCMV6-Entry vectors (Origene, Rockville, MD), Boyden's chambers were from Neuro Probe, Inc. (MD, USA).

### Cell lines and culture conditions

Cell lines and culture conditions employed in this study, including cell culture under controlled conditions of hypoxia, are as previously described [10,39]. HepG2 and Huh7 cells (American Type Culture Collection, USA) were maintained in Dulbecco's modified Eagle's medium supplemented with 10% fetal-bovine serum, 100 U/ml penicillin, 100 μg/ml streptomycin and 25 μg/ml amphotericin-B. To evaluate the role of hypoxia, cells were seeded in normoxic conditions to obtain the desired sub-confluence level (65-70%) and then incubated in strictly controlled hypoxic conditions (3% O_2_) up to 96 hours, as previously reported [2,3]. Cellular extracts and the corresponding medium were tested for SERPINB3 protein content by ELISA (SCCA-LISA, Xeptagen, Marghera, Italy) using recombinant SERPINB3 protein for calibration curve (range 0.02-200 ng/ml). In some experiments, we also employed HepG2 stably transfected with the human SERPINB3 cDNA in order to over-express SERPINB3, or with the empty vector alone as control, obtained as previously described [1].

### Generation and selection of HepG2 cells stably overexpressing HIF-1α or HIF-2α

The pCMV6-based mammalian expression vectors, empty (used as a control) and encoding HIF-1α or HIF-2α (OriGene, Rockville, MD) were propagated into E. coli (JM109 strain) and purified using Plasmid DNA purification NucleoBond XtraMIDI (Macherey-Nagel, Germany). HepG2 cells were seeded and then transfected 24 hr later with 10 μg of each vector using Lipofectamine 2000 (Invitrogen, Carlsbad, CA). Transfected cells were subsequently cultured in normal medium supplemented with 400 μg/ml G418 to specifically get rid of non-transfected cells until the growth rate of transfectants paralleled that of non-transfected controls. HIF-1α and HIF-2α expression of the generated stable transfectants was carefully characterized, after which the cell lines carrying the empty vector (H/pCMV6) and overexpressing either HIF-1α (H/1α) or HIF-2α (H/2α) were then used for the experiments described.

### Quantitative real-time PCR (Q-PCR)

RNA extraction, complementary DNA synthesis, quantitative real-time PCR (Q-PCR) reactions were performed as previously described [30]. SERPINB3 and HIF-2α mRNA levels were measured by Q-PCR, using the SYBR® green method as described [30]. The amplification mix was prepared using Roche LightCycler FastStart DNA MasterPLUS SYBR Green I kit following manufacturer's instructions and real-time PCR was performed using LightCycler instrument. Oligonucleotide sequence of primers used for RT-PCR were: sense, 5′-GCAAATGCTCCAGAAGAAAG-3′, reverse 5′-CGAGGCAAAATGAAAAGATG-3′ (for human SERPINB3); sense, 5′-GGAGCCACGGTCTCTCAGTA-3′, reverse 5′-TGCATCTATGGGGATGAGAA-3′ (for human SERPINB4); sense, 5′-CGCTAGACTCCGAGAACAT-3′, reverse 5′-GGCTTGAACAGGGATTCAGT-3′ (for human HIF2α). Gliceraldehyde-3-phosphate dehydrogenase (GAPDH) was used as internal reference and co-amplified with target samples using identical Q-PCR conditions. In particular, expression of SERPINB3 and HIF-2α mRNA was also assessed by Q-PCR in 67 frozen liver tumor samples displaying different patterns of SERPINB3 expression (SERPINB3 negative/low expression or SERPINB3 high expression) [30]. Samples were run in triplicate and mRNA expression was generated for each sample. Specificity of the amplified PCR products was determined by melting curve analysis and confirmed by agarose gel electrophoresis.

### Chromatin immuno-precipitation (ChIP)

ChiP and ChIP-PCR were performed as described with few modifications [50]. Adherent HepG2 cells were treated with 1% formaldehyde for 10 min at 37 °C and cross-linking stopped by adding glycine at 0.125 M final concentration. Cells were washed twice using ice-cold PBS containing protease inhibitors: 1 mM phenyl-methyl-sulphonyl-fluoride (PMSF), 1 μg/ml aprotinin and 1 μg/ml pepstatin-A. Cells were harvested with 200 μl (every 10^6^ cells) of lysis buffer (50 mM Tris–HCl, pH 8.1, 10 mM EDTA, 1% SDS, 1 mM PMSF, 1 μg/ml aprotinin and 1 μg/ml pepstatin-A) and lysis performed by incubation for 10 min on ice. Cell lysates were then sonicated (80 pulses, 15 sec on and 28 sec off each, at maximum power in a Sonoplus apparatus; Bandelin GM3200) to generate DNA fragments of 100-500 bp. After centrifugation (13000 rpm, 10 min, 4 °C), the supernatant was 10-fold diluted with ChIP dilution buffer (16.7 mM Tris–HCl, pH 8.1, 167 mM NaCl, 1.2 mM EDTA, 0.01% SDS, 1.1% Triton X-100) and 4 % of this sample recovered and used as an indicator of chromatin content in each sample (input). In a new tube 2 μg antibody was incubated with 14 μl Protein G-Dynabeads (#100.03D; Life Technologies Italia, Monza, Italy ) and 200 μl PBS/Tween-20 0.02%, for 1 hour at RT under constant agitation. The bead-antibody complexes were recovered with a Dyna magnet (Life Technologies Italia, Monza, Italy), resuspended in 100 μl PBS/BSA 100 mg/ml and added to each sample to be incubated for 16-18 hours at 4 °C under constant agitation. In parallel, each sample was subjected to the same procedure without antibody (negative control). Antibody-protein-DNA complexes were transferred on the magnet and supernatant was removed. After extensive washing, complexes were eluted from beads with 500 μl elution buffer (0.1 M NaHCO3, 1% SDS). Following addition of 0.2 M NaCl, all samples, including input, were incubated for 4 h at 65 °C to revert cross-linking. After treatment with 10 μM RNAase and digestion with 40 μM proteinase-K, DNA was extracted using QIAcquick PCR purification, according to the manufacturer's recommendations (Qiagen, #28106) and DNA eluted in 40 μl of elution buffer. ChIP-grade antibodies used were: rabbit anti-HIF-2α (Novus Biologicals, NB100-122); mouse anti-RNApol II (Abcam, #ab5408). Other antibodies used were: rabbit IgG (Sigma-Aldrich, #G5518) and mouse IgG (#M7023) from Sigma-Aldrich. Immunoprecipitated DNA was quantified by Quantitative Real-Time PCR (Q-PCR). Q-PCR (2 min at 50°C, 5 min at 95 °C, 45 cycles at 95 °C for 15 sec and 60 °C for 1 min) was performed with 2 μl of eluted DNA using the ABI Prism 7500 Sequence Detection System (Applied Biosystems; Carlsbad, CA, U.S.A.) and Power SYBR® Green PCR master mix (Applied Biosystems). A melting curve analysis was performed to discriminate between specific and non-specific PCR products. The relative amount of immunoprecipitated SERPINB3 promoter DNA was determined using the following primers: for 5′-GGAATGATGTACTGATCCATGC-3′'; rev 5′-TACTCGAGACCCTGGAAACC-3′. Data were normalized by input DNA and expressed with respect to those of control IgG (used as calibrator). The amount of immunoprecipitated SERPINB3 promoter DNA was also monitored by RT-PCR using the same primers indicated for Q-PCR.

### HIF-1α and HIF-2α silencing by small RNA interference

RNA interference experiments to knockdown HIF-1α or HIF-2α expression in HepG2 cells were performed using siRNA duplex (Qiagen Italia, Milano, Italy) as previously described [10]. The following target sequences were used:

HIF-1α: 5′-AGGAAGAACTATGAACATAAA-3′;

HIF-2α: 5′-CCCGGATAGACTTATTGCCAA-3′.

The siRNAs and related non-silencing controls were transfected in HepG2 cells with lipofectamine 2000 transfection reagent (Life Technologies Italia, Monza, Italy) according to manufacturer's instructions up to 72 hrs. Transfected cells in fresh medium were then exposed for further 48 hrs to the desired experimental conditions and then harvested for sample preparation.

### Western Blot analysis

Total cell lysates or nuclear vs cytosolic extracts, obtained as described, [10,39] were subjected to sodium dodecyl sulfate-polyacrylamide gel-electrophoresis on 12%, 10% or 7.5% acrylamide gels, incubated with desired primary antibodies, then with peroxidase-conjugated anti-mouse or anti-rabbit immunoglobulins in Tris-buffered saline-Tween containing 2% (w/v) non-fat dry milk and finally developed with the ECL reagents according to manufacturer's instructions. Sample loading was evaluated by reblotting the same membrane with β-actin antibody.

### Patients and samples

In the present study we employed HCC specimens obtained under written informed consent at the time of surgery in 67 patients with cirrhosis of different etiology. Clinico-pathological and molecular characteristics of these specimens as well as follow-up procedures applied to these patients were extensively described in a previous study [30]. All the patients underwent surgical resection as first-line therapy without pre-operative anticancer treatment and distant metastases. Tumour tissue samples were collected and part was formalin fixed and paraffin embedded, whereas the remaining part was immediately frozen at −80 °C for transcript analysis. A series of 18 paraffin-included liver specimens from cirrhotic HCV patients (METAVIR F4) carrying HCC (G1 and G2, Edmonson-Steiner grading) was also included in the study for immuno-histochemistry analysis.

The use of human material conforms to the ethical guidelines of the 1975 Declaration of Helsinki and was approved for this study by the Bioethical Committees of University of Torino and of Padova.

### Immuno-histochemistry

Immuno-histochemistry (IHC) was performed on paraffin liver sections of HCC specimens derived from patients with hepatitis C virus (HCV)-related liver cirrhosis (METAVIR F4). The use of human material conforms to the ethical guidelines of the 1975 Declaration of Helsinki and was approved for this study by the University of Torino Bio-ethical Committee. Immuno-staining procedure was briefly described: paraffin sections (2 μm thick), mounted on poly-L-lysine coated slides, were incubated with polyclonal antibody raised against the C-terminus of human HIF-2α protein (Novus Biologicals Inc.; dilution 1:100) or against heme oxygenase 1 (HO-1, Stressgen, Vancouver, BC, Canada; dilution 1:100), as previously described [7], or the monoclonal antibody against SERPINB3 (Santa Cruz Biotechnology, CA, USA; dilution 1:50). After blocking endogenous peroxidase activity with 3% hydrogen peroxide and performing microwave antigen retrieval, primary antibodies were labeled by using EnVision, HRP-labeled System (DAKO) and visualized by 3′-diaminobenzidine substrate. For negative controls the primary antibodies were replaced by isotype- and concentrations-matched irrelevant antibody.

### Detection of intracellular generation of ROS

Cultured cells, seeded in 12-well culture plates (10^5^ cells/well), were either exposed to hypoxia or treated or not with 50 μM H_2_O_2_ under normoxic conditions for 15 min. ROS generation was detected as the conversion of 2′,7′-dichlorodihydrofluorescein diacetate (DCFH-DA, 5 μM) into the corresponding fluorescent derivative. Cells were observed and photographed under a Zeiss fluorescence microscope [2,6,7]. ROS generation was also detected by combining DCFH-DA technique and flow cytometric analysis. Cells were seeded in P35 dishes (5×10^5^ cells/dish), cultured for 24 hrs and exposed to hypoxia alone or hypoxia plus 2.5 μM rotenone for 15 min before addition of 5 μM DCFH-DA. Cells were rapidly washed with PBS, collected by trypsinization and re-suspended in PBS for analysis. Detection of DCF green fluorescence (FL1) was performed on at least 5,000 cells per sample with a FACScan equipped with a 488 nm argon laser using the CellQuest software (Becton-Dickinson, Milano, Italy). The peak of FL1 intensity of DCFH-DA-stained control cells grown under normoxic conditions was set to channel 101 and retained for all measurements. As a positive control, cells grown under normoxic conditions were treated with 50 μM H_2_O_2_ for 15 min, stained with DCFH-DA as above and assayed during the same analytical session.

### Invasion assay

Invasion assay was performed as previously detailed ^[10]^ by employing Boyden chambers equipped with 8-μm porosity polyvinylpyrrolidone-free polycarbonate filters coated with 50 μg/ml of Matrigel solution for invasion assay. Invasiveness was quantified by counting, with a Zeiss microscope (Oberkochen, Germany) equipped with bright-field optics (x40), crystal violet stained cells that invaded Matrigel. For each condition, cells were counted in ten randomly chosen fields, averaged as means ± SEM and expressed as number of invading cells per high-power field.

### Statistical analysis

For cell culture experiments data in bar graphs represent means ± SEM, and were obtained from average data of at least three independent experiments. Luminograms and morphological images are representative of at least three experiments with similar results. Statistical analysis for these experiments was performed by Student's t-test or ANOVA for analysis of variance when appropriate (p < 0.05 was considered significant). Statistical analysis designed to assess the association of SERPINB3 with HIF-2α expression in liver tumors of surgically resected patients were performed by non-parametric procedures using the Mann Whitney test. Moreover, in order to evaluate simple linear relationships between quantitative variables, Spearman's correlation coefficient was applied. All tests were two-sided. The calculations were carried out with GraphPad InStat Software (San Diego, CA). The null hypothesis was rejected at p < 0.05.

## SUPPLEMENTARY MATERIAL, FIGURES


