# Supplementary material for: Hypoxia up-regulates SERPINB3 through HIF-2α in human liver cancer cells
Source: Oncotarget. 2014 Dec 10;6(4):2206–21. doi: 10.18632/oncotarget.2943 (PMC4385846; doi:10.18632/oncotarget.2943)
Supplement: Supplementary file 1 [file oncotarget-06-2206-s001.pdf]

Hypoxia up-regulates SERPINB3 through HIF-2α in human liver cancer cells

Supplementary Material

SERPINB3 (ex SCCA-1)
GENE BANK AB034984

```
   1 ttttattagt gatttcaaaa ggagagggag tgtacgaata gagtgtgggt cacagagatc
  61 acgtgcttca caaggtgata gaatatcaca aggcaaatgg aggcagggtg agatcacagg
 121 accacaggac ctgggtgaaa ttaaaattgc taatgaagtt tcgggcacgc attgtcattg
 181 ataacatctt atcaggagac agggtttgag agcagacaac cggtctgacc aaaaatttat
 241 tagatgggac tttcctcatc ctaataagcc taggagcgct acgggaggtt ggggcttatt
 301 tcatccctac agcttcaacc ataaaagacg gctgcccccc aaagcggcca ttttaaaggc
 361 ctaccctcag gggcatattc tctttctcag ggatgttcct tgatgagaaa aagaattcag
 421 cgatatttct cccatttgct tttgaaagaa gagaaatatg gctctgttcc acccagctca
 481 ccggcggtca gagtttaagg ttatcactca tgttccctga acattgctgt tatcctgttc
 541 tttttcaag gtgcccagat ttcatattgt tcaaacacac atgctctaca aataatttat
 601 gcagttaatg caatcatcac agggtcctgg gaccacatac attctcctca gcttgcaaag
 661 atgatgggat taagagatta aagtaaagac aggcatagga aatcacaagg gtattgattg
 721 aggaagtgat aagtgttcat gaaatcttca caatttatgt tcagagattg cagtaaagac
 781 aggcataaga aattataaaa gtattaattt ggggaactaa taaatgtcca tgaaaacttc
 841 ataatctatg ttcttctgcc atggcttcag ccagtccctc ggttcagggt ccctgacttc
 901 ctgcaacata catgtgagac tatttcccgc tctgcttttc aaaccttact ggagttgttt
 961 tccctcatga aaactaagaa aggaaagcta gttaatctta ttctgaggtt gttcaatata
1021 tacatattca catctgtaga aagatccttg ggaatacagt aattggcata tattctgtta
1081 tttgatgctt gaaaaatctc ttccactaac cagtttccct atagataggc acaagcacat
1141 aggtaagaaa caataaataa atgttctctt taatttgtaa cttcacaatg ctgagaaaac
1201 tttacagcct tcataaggaa gtgaggtcca ggaaaatcta ggagatattt gttaaccaac
1261 ctataaagac attagtaatg acaggatatt tcctgaaagt gtaatttccc attgaggatt
1321 tgtttttaat ttctggattc ctggatccaa tgaagttggc ataggtttat gaaatgccaa
1381 gatacataag ttggcaagtg ttcacatgca aaaaacttct tggaattcct gagctctctg
1441 tggcaatata tgacatcagg atatgtccca tctcgcacat caggatatgt cctgtcaaga
1501 atgtctatca catgccagga gtacttttta ggaacagaaa aaaatgtctg aaatggtttc
1561 tcatttgaac tcatccaagc tttctctaaa tttaagcaaa ctcctggtca ttttcagtta
1621 gtacctttcc tcaagttcaa ccttcatgac aaacctcagc atctcagaag atttagccat
1681 agtctgaaat tctcttccat agactggtcc cctgtaatcc cagtttgcct cagcttgtta
1741 tcctgctttt tattcccctc tattcccagg ctgagcttct tgcttctgtc ctatgagacg
1801 ttagattcct tcactttggt acccaagtaa acccatcctt ctccatatac aggaaggtcc
1861 atttttctct tacagccctg gatgcagact cagctaagaa gaccattatt cattttttgga
1921 attcttcatc taggatattt cctcttgttt ctttctctcc tatctttgag cttttttagat
1981 catcaacacc ccattagtct attacccaac ttaaatcagg gaacttatac ctcccaaact
2041 cattcagaga ctccaaacat atatattgat acaggagacc taagaagagc atgtcttggg
2101 ggttgaggaa acaggcaggt gagaaacttc cagattggaa acacagcttc ctttctcccg
2161 tccagcccct acttcatcct atctgtttcc ggaaccttgt tgtagatgaa tctcccttga
2221 cttcatgatg tgctgagaaa acaaactcat ggctggtgtt aaaaagggcc catgacaata
2281 ccaagtgttg gggagaatgt ggagaaatca gaactctatt cacagtcggt tggaatgcac
2341 acttgtgcag aattctatgg agaagagtct ggcatttcct caaaatgtta acctggattt
2401 accatatgac ccagcgattt cattcatagg tttatactca aaagaaatga agaaatatgc
2461 catgcaaaaa aatgtacatg aaaagtcaaa acatcattat tcataatagt aaatggatgg
2521 aaacaacaca aatgtccatc aacttatgaa taaagaaaat gtggtctatt catagaatgg
2581 aatattattc gaccacaaaa aggaatgatg tactgatcca tgcaatgacg tggacaaacc
2641 ttgaagataa tactagatga aagaagccag tcacaaaagg acttactgta tgattcgatt
2701 tacgtgaaat gtttagaata ggcaaatcca tagaaacagg aggtagatta ctggtttcca
2761 gggtctcgag taagggaaga acgagataca agttttcttt tggaggtagt gaaattgttg
2821 tggaacgaga tcatgatggt gatagcacaa ctttgtgaat ataataaaat cattgaattg
2881 tacagttgaa tttgtggcat ataaattata tgttaaaaaa gggggtccac aaaacaaaca
2941 gccccccact ctggttgtca gggagatatt ggattaaatg gccttggaca acaaccctct
3001 ccctggccac agacattctt cagattacaa gatattccag aggaaacact ggaatgagtc
3061 tgaagccagg tgctaaatgg aaggaccacc aagaaacgtt gtgatcctga caggtcaagc
3121 aacttctttt tctgcttaat ttttaaatga aaaattagaa agctgacatt caaaatggcc
3181 cgtctgtttc aattgctctt ctcagtgtca gcctgttaac tcaatgtgtt agtctgtttt
3241 catgctgctg ataaagacat acctgagatt aggaagtaaa agaggtttaa ttggacttag
3301 agttccacgt gattggggag gcctcagaat cacggtagga ggcaaaagtt attcttacat
3361 ggtggctgca agagaaaatg aggaagaagc aaaagaagaa accccctaata aacccattgg
3421 atctcctgag acttattaac tatcatgaga atagcacaag aaagaccggc ccccatgatt
3481 caattacctc tacctgggtc cctccaacaa catgtggaaa ttctggtaga tacaattcaa
3541 gttgagattt gggtgggaac acagccaaac catatcactc agcaaggcag ataactttct
3601 cactgagcct atgcaacaga aaaccatctg ggatggttgt aaggggcaca ggaagtgact
3661 ggtaggatca ctgccaaagc tgagcattca ggagaaggca atagaatcct attctccata
3721 gtatgctata agatactgaa gtacacttct tcactatctc tttggactta gaattagcac
3781 tatattcctt gttatacaga aaaattacta aggaaattca taggatgaca aaaactttca
3841 gaactgaaaa acaggaaatg taagcttttt agttctttgg tattcgaagt atgcctaaaa
3901 gacaatgcaa aatccaagaa aagaatggtg gggtttttgt ttgtttgttt ttgtttttgt
3961 tttacagctg gagtagaata caaagggatg gagttgaaac aaatgagagg aaattggaat
4021 tctaaactta ttctcattgg cattagaaag gcacctacat gtatttcaca tgagccggtg
4081 actgctgact tgcattctta tttttccct atagattaaa aaggaggtac aatggtagaa
4141 ctgtaatcct gtcctttgtc ataaattttc gtattcataa aggtgagtgt tagcccgctt
4201 gtgaaatctg aagttgagta acttcaaata ctaaccacag agggagaggc agcaagagga
4261 gaggcataaa ttcaggatct caccccttcat tccacagaca cacatagcct ctctgcccac
4321 ctctgcttcc tctaggaaca caggtaagag cttcaagcct ctccagctta ataacatgaa
4381 ttatttttga gaataataat gatactgtgt tctatatcat gcatctcctg cattctgtct
4441 gattatattt tacttattct gccagagcaa aattaaaata cctatttcat ctgatttgtc
4501 ctttatctaa attgcttagt tccaagtaaa ccaaggcact tttaggaaca cagagggaga
4561 gtgccttgca gccagagagt cttgaaggag atgtcaggga cgcatcttaa cagctggttg
4621 gatgtgatcc acagaggtct cctgttagca ttcattgtaa agccttccta cctagcccta
4681 gtgtagccag caatgaagga aagagggtct attacttatt tacagtagtc tttaaaaaca
4741 ctaattttgt gaggcttcta attaagacat taatatattt aatatatgca cattgtagaa
4801 agattgaaac gttaaaaata agatgaggaa aactttaaat gtcaaaatct cacaacacag
4861 atatataatt tctttaagaa aattgtacta caaaatacca ttccatttat taaagtcatt
4921 ctgacaggaa tctgatgctt ttccaggagt tccagatcac atcgagttca ccatgaattc
4981 actcagtgaa gccaacacca agttcatgtt cgacctgttc caacagttca gaaaatcaaa
5041 agagaacaac atcttctatt ccccctatcag catcacatca gcattaggga tggtcctctt
5101 aggagccaaa gacaacactg cacaacagat taagaaggta gctatcagca tcattatgtt
5161 gtcctgttgc agtttttctc tggttccgtc ggctagcacg cagatggtaa tagatgtggt
5221 ggtctgatgg gtagcacagg gggctgagca ggaattcccg taactgtgag accactgg..
```

Suppl. Figure 1: *SERPINB3 promoter map.* The promoter map showing two ACGTG sequences (i.e., containing the consensus HRE sequence CGTG, in green) as well as four GGAC sequences and one CGGA sequence (in brown) that have both been proposed as ETS binding sites able to increase HIF-2α binding affinity. CATAAA sequences (in red), box transcription start (green) and the region amplified with the primers designed for ChIP (blue) are highlighted.

:A

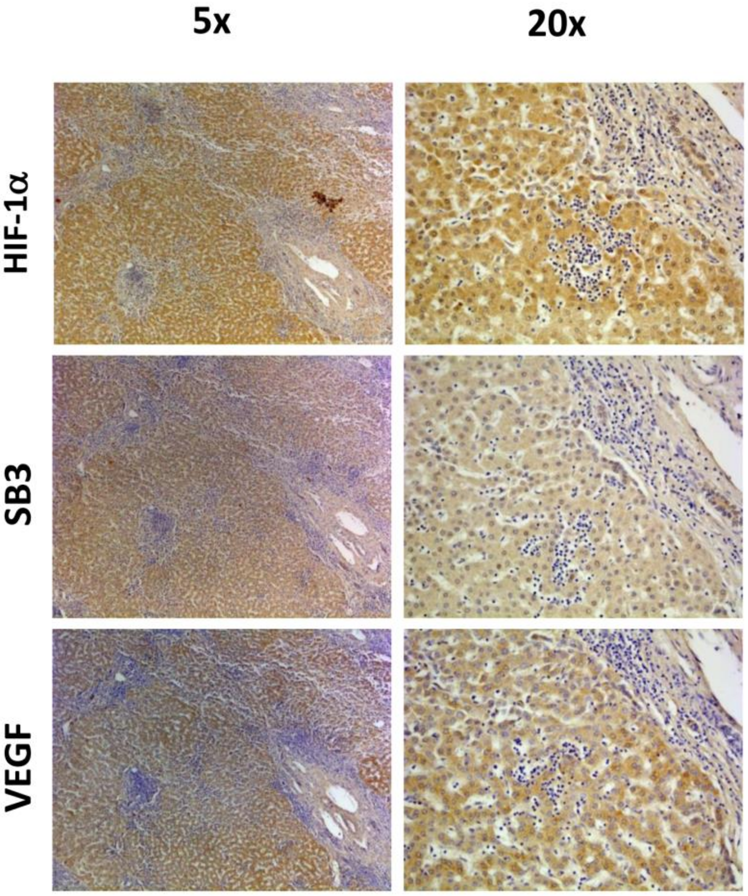

Human HCC in HCV cirrhotic patient - G1

:B

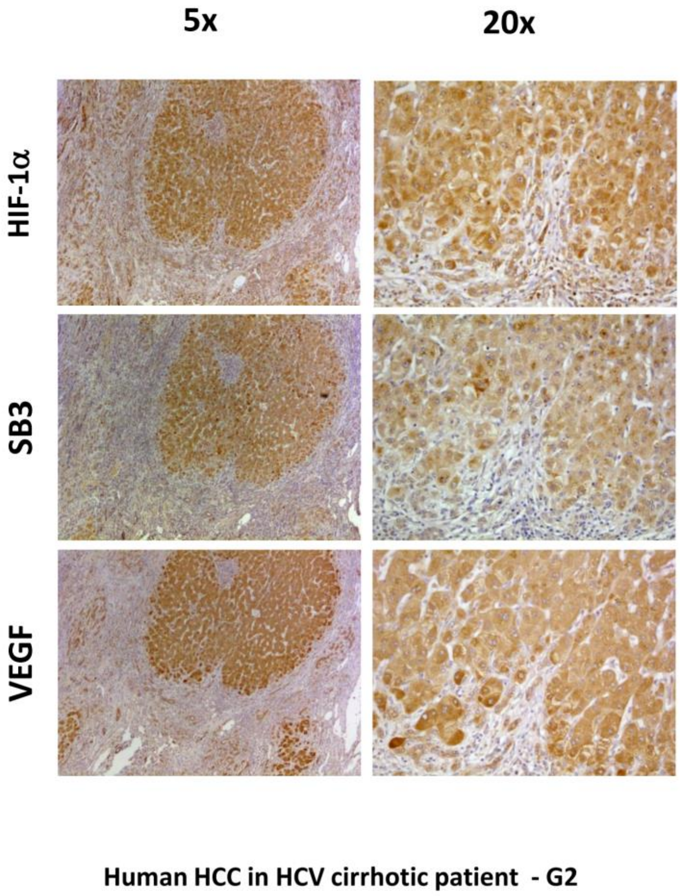

Suppl. Figure 2A,B: ***HIF-1α, SERPINB3 (SB3) and VEGF expression in human HCC.*** Immune-histochemistry analysis for HIF-1α, SERPINB3 (SB3) and VEGF-A performed on 2 μm thick serial sections obtained from representative, grade G1 **(panel A)** or G2 **(panel B)** human HCC developed in HCV cirrhotic patients. Immune positivity for SERPINB3 is evident in the areas/cells showing immuno positivity also for HIF-1α and VEGF. Original magnification as indicated.

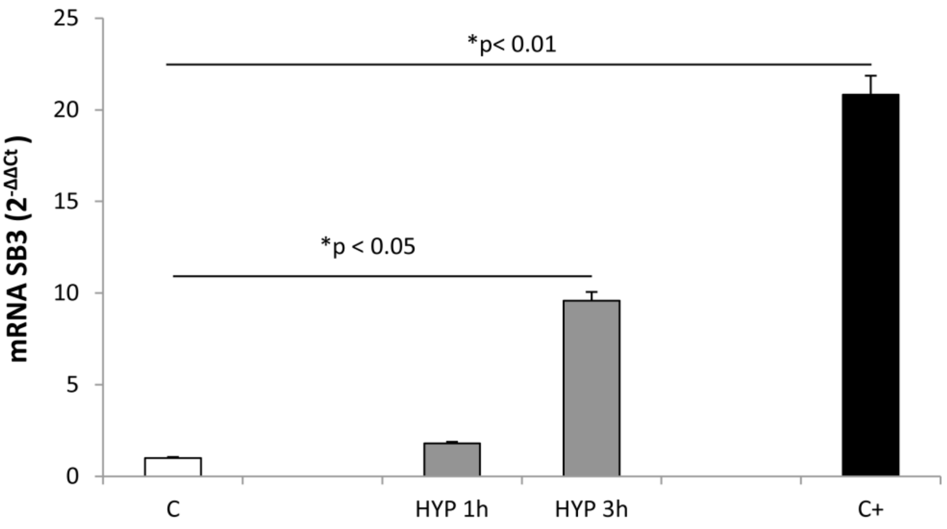

Suppl. Figure 3: ***SERPINB3 expression at 0.1% oxygen.*** Representative data of a time-dependent analysis of SERPINB3 (SB3) transcripts quantified by Q-PCR in HepG2 cells not exposed (control, C) or exposed to severe hypoxic conditions (HYP 0.1%) for 1h or 3hrs. (*p< 0.05 vs control values of SB3; **p< 0.01 vs control values of SB3) .

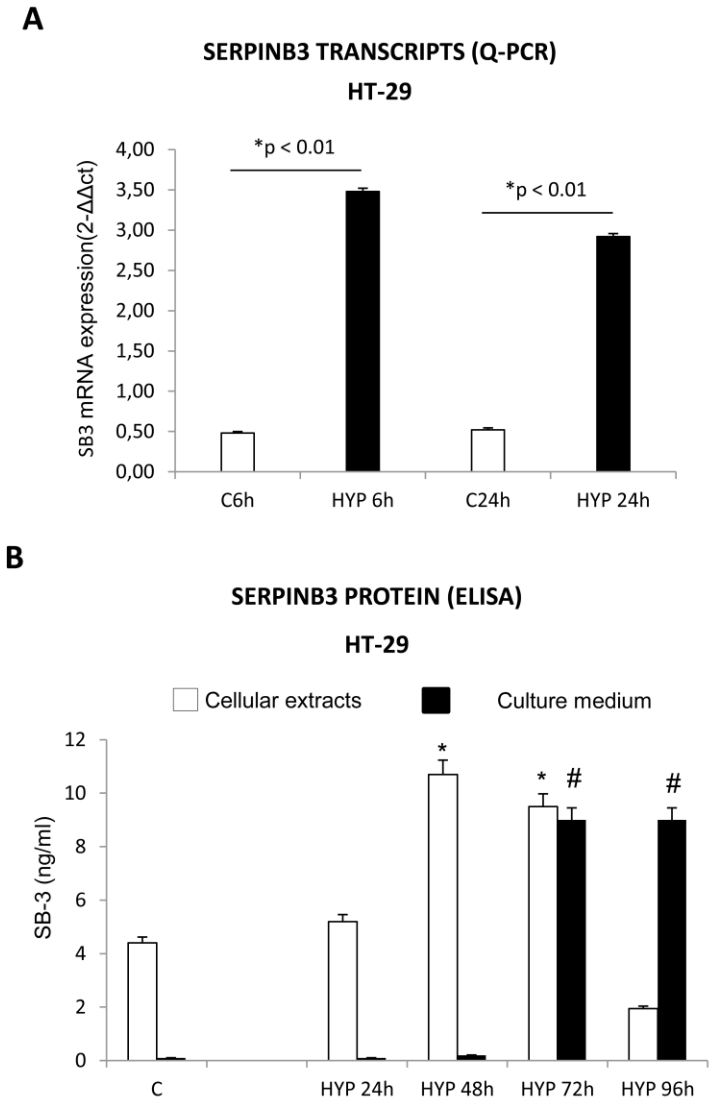

Suppl. Figure 4: ***Hypoxic conditions up-regulate SERPINB3 (SB3) expression in HT-29 cells.*** **Panels A**. Time-dependent analysis of SERPINB3 transcripts by quantitative real-time PCR (Q-PCR) in HT-29 cells not exposed (control, C) or exposed to hypoxic conditions (HYP) for the indicated times (*p< 0.01 vs control values). **Panels B**. Time-dependent analysis of SERPINB3 protein levels (ng/ml) by ELISA in cellular extracts (white columns) or culture medium (black columns) of HT-29 cells not exposed (control, C) or exposed to hypoxia (HYP) for the indicated times. Data are expressed as means ± SEM of three independent experiments (*p< 0.01 vs control values of SB3 in cellular extracts; $^{\#}$p< 0.01 vs control values of SB3 released in culture medium).

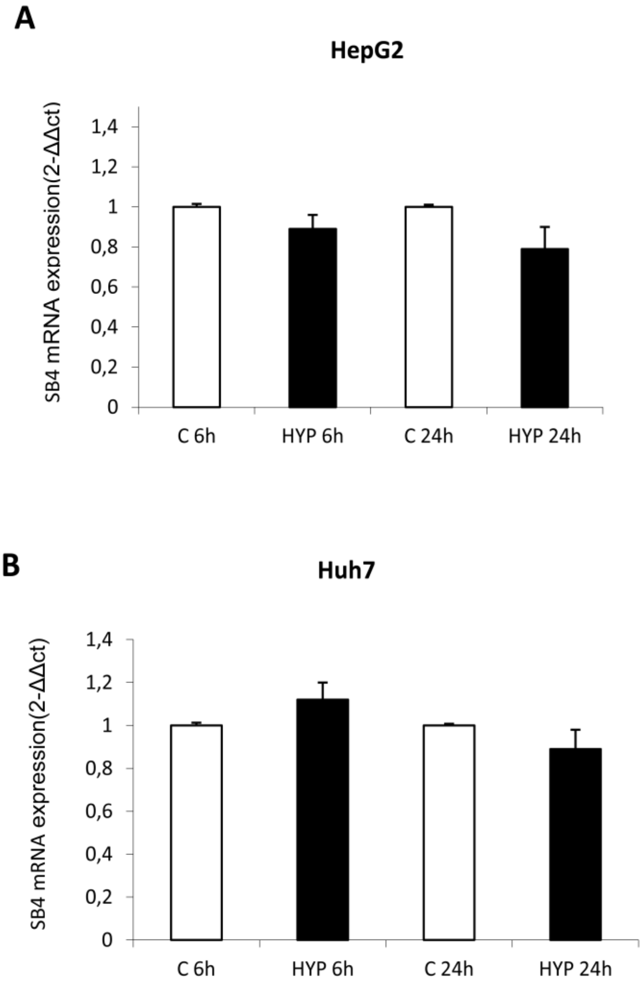

Suppl. Figure 5: ***Hypoxic conditions do not induce up-regulation of SERPINB4 (SB4).*** **Panels A,B**. Time-dependent analysis of SERPINB4 transcripts by quantitative real-time PCR (Q-PCR) in HepG2 (panel A) or Huh7 (panel B) cells not exposed (control, C) or exposed to hypoxic conditions (HYP) for the indicated times.

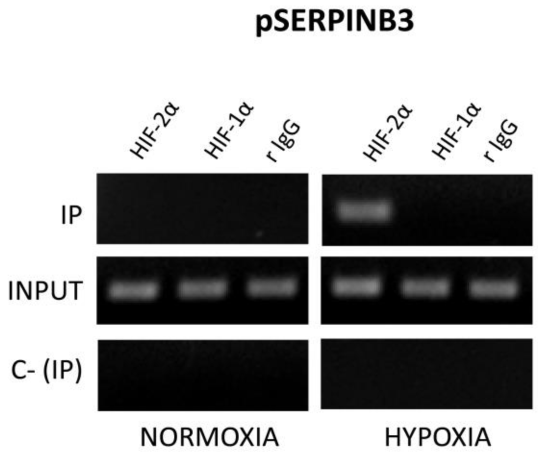

Suppl. Figure 6: ***HIF-1α does not bind to SERPINB3 promoter.*** HepG2 cells were incubated for 3 hrs in normoxia or hypoxia and lysed. Lysates were subjected to ChIP using antibodies against HIF-2α, HIF-1α or control IgG (rabbit, r). RT-PCR for SERPINB3 promoter (pSERPINB3) was performed with IPed DNA after IP or using input DNA (INPUT). C- (IP) was a negative control in the absence of antibody. Images obtained in one experiments out of three which provided similar results.

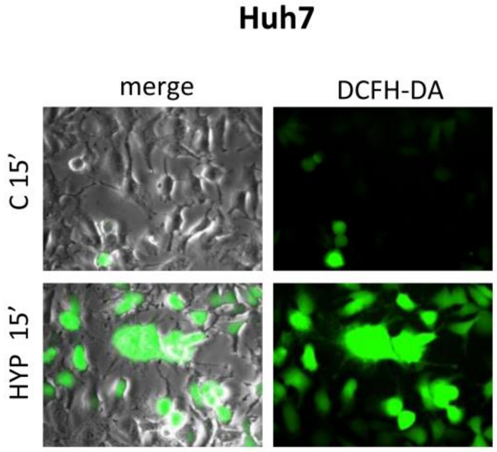

Suppl. Figure 7: ***Hypoxia up-regulates intracellular ROS levels in Huh7 cells.*** Representative images documenting early detection of intracellular ROS (DCFH-DA probe) generation in Huh7 cells not exposed (control, C) or exposed to hypoxia (HYP) for 15 minutes, as detected using fluorescence microscopy. DCFH-DA fluorescence (right panels) vs merging of fluorescence and phase contrast images (left panels).

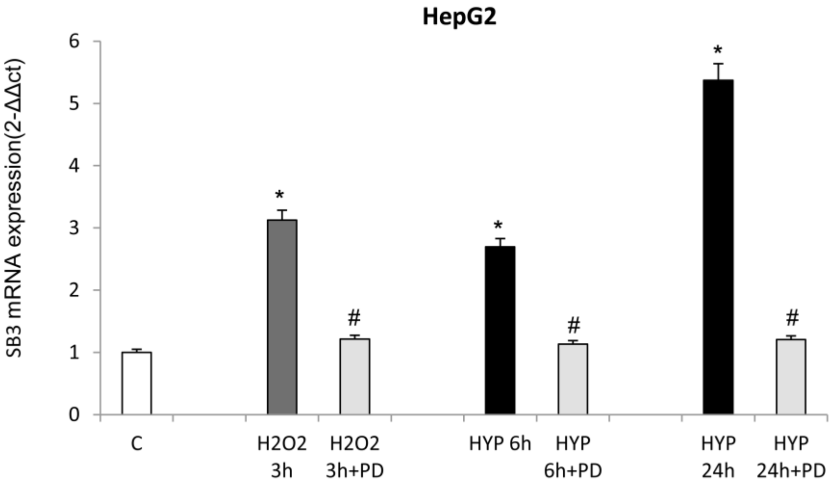

Suppl. Figure 8: ***Ras/Erk signaling is involved in the activation of SERPINB3 gene expression in the context of hypoxia and ROS generation.*** Analysis of SERPINB3 transcripts by quantitative real-time PCR (Q-PCR) in HepG2 cells not exposed (control, C) exposed to H_2O_2 50µM or exposed to hypoxic conditions (HYP) for the indicated times. In order to investigate ERK involvement HepG2 cells were pre-treated (or not) with the specific MEK pharmacological inhibitor PD98059 30 µM (*p< 0.01 vs control values of SB3; # p< 0.01 vs related treatment condition, respectively).

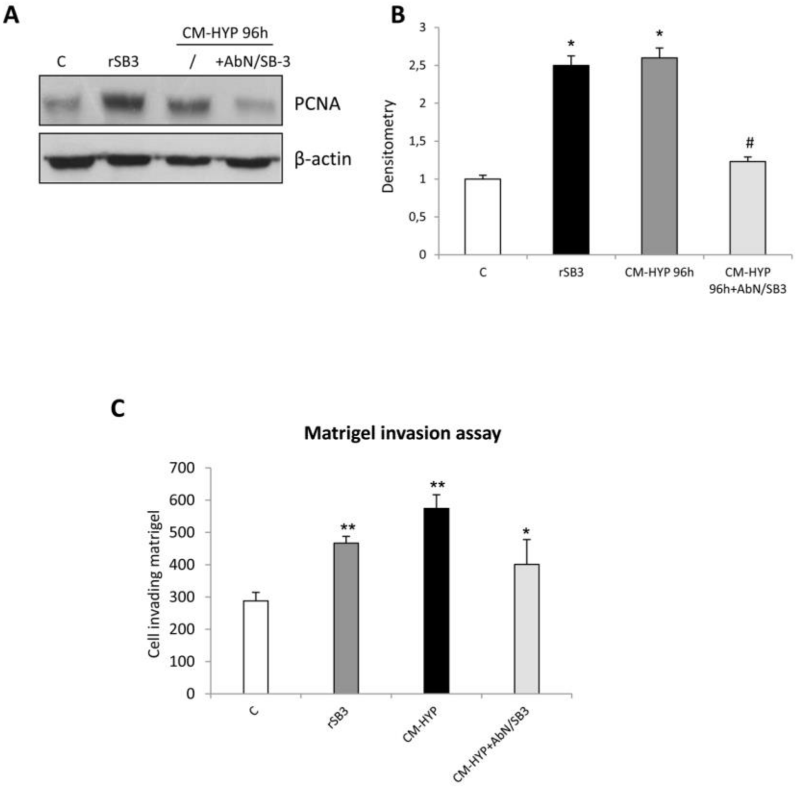

Suppl. Figure 9: Analyses of the possible role of SB-3 released during hypoxic conditions. Panel A. WB analysis of PCNA protein levels on total extracts obtained from HepG2 cells maintained in normoxic conditions not exposed (control, C), treated with human recombinant SB3 100 ng/ml (rSB3) or treated with conditioned hypoxic medium collected by HepG2 cell maintained in hypoxia up to 96 hrs (CM-HYP 96h) in the absence or not of a neutralizing antibody against SERPINB3 (CM-HYP 96h+AbN/SB3). Equal loading was monitored by reblotting membranes for β-actin. **Panel B.** Matrigel invasion assay (Boyden's chambers) performed by employing HepG2 cells maintained in normoxic conditions not exposed (control, C), treated with human recombinant SB3 100 ng/ml (rSB3) or treated with conditioned hypoxic medium collected by HepG2 cell maintained in hypoxia up to 96 hrs (CM-HYP 96h) in the absence or not of the neutralizing antibody against SERPINB3 (CM-HYP 96h+AbN/SB3). Data are expressed as means ± SEM of three independent experiments (**p< 0.01 vs control values, *p< 0.05 vs related treatment condition, respectively).
